# Identification and Characterization of a Novel *Robigovirus* Species from Sweet Cherry in Turkey

**DOI:** 10.3390/pathogens8020057

**Published:** 2019-04-27

**Authors:** Kadriye Çağlayan, Vahid Roumi, Mona Gazel, Eminur Elçi, Mehtap Acioğlu, Irena Mavric Plesko, Jean-Sebastien Reynard, Francois Maclot, Sebastien Massart

**Affiliations:** 1 Plant Protection Department, Agriculture Faculty, Mustafa Kemal University, 31034 Hatay, Turkey; caglayan@mku.edu.tr (K.C.); monagazel@hotmail.com (M.G.); mehtappathem@hotmail.com (M.A.); 2 Plant Protection Department, Faculty of Agriculture, University of Maragheh, 55181 Maragheh, Iran; 3 Plant Production and Technologies Department, Faculty of Agricultural Sciences and Technologies, Nigde Omer Halisdemir University, 51240 Nigde, Turkey; eminur@gmail.com; 4 Agricultural Institute of Slovenia, Hacquetova 17, SI- 1000 Ljubljana, Slovenia; irena.mavricplesko@kis.si; 5 Virology-Phytoplasmology Laboratory, Agroscope, 1260 Nyon, Switzerland; jean-sebastien.reynard@agroscope.admin.ch; 6 Plant Pathology Laboratory, TERRA-Gembloux Agro-Bio Tech, University of Liege, Passage des Deportes, 2, 5030 Gembloux, Belgium; francois.maclot@doct.ulg.ac.be (F.M.); sebastien.massart@uliege.be (S.M.)

**Keywords:** *Prunus avium*, high throughput sequencing, *Betaflexiviridae*, cherry virus Turkey

## Abstract

High throughput sequencing of total RNA isolated from symptomatic leaves of a sweet cherry tree (*Prunus avium* cv. 0900 Ziraat) from Turkey identified a new member of the genus *Robigovirus* designated cherry virus Turkey (CVTR). The presence of the virus was confirmed by electron microscopy and overlapping RT-PCR for sequencing its whole-genome. The virus has a ssRNA genome of 8464 nucleotides which encodes five open reading frames (ORFs) and comprises two non-coding regions, 5′ UTR and 3′ UTR of 97 and 296 nt, respectively. Compared to the five most closely related robigoviruses, *RdRp*, *TGB1*, *TGB2*, *TGB3* and *CP* share amino acid identities ranging from 43–53%, 44–60%, 39–43%, 38–44% and 45–50%, respectively. Unlike the four cherry robigoviruses, CVTR lacks ORFs 2a and 5a. Its genome organization is therefore more similar to African oil palm ringspot virus (AOPRV). Using specific primers, the presence of CVTR was confirmed in 15 sweet cherries and two sour cherries out of 156 tested samples collected from three regions in Turkey. Among them, five samples were showing slight chlorotic symptoms on the leaves. It seems that CVTR infects cherry trees with or without eliciting obvious symptoms, but these data should be confirmed by bioassays in woody and possible herbaceous hosts in future studies.

## 1. Introduction

The world production of cherries has reached about 2.3 million tons and is mainly distributed in Asia (43%), Europe (37%) and America (18%) [1]. Turkey is the first producing country (494,325 tons) and the centre of domestication of sweet cherry. The high genetic diversity of cultivars is an asset for developing new cultivars [2]. In addition, due to intensive and long lasting cherry production, the diversity of pathogens in Turkey might be higher than in other parts of the world. This is therefore an appropriate region for characterizing the virome of this plant species. 

To date, 44 viruses and three viroids have been described in the nine main cultivated *Prunus* species. Within the last 5 years, seven of these viruses and one viroid have been discovered in *Prunus* hosts among which four were described on cherries [3]. This number is constantly increasing and includes viruses belonging to genera that were previously unknown to infect *Prunus* species [4,5]. High throughput sequencing (HTS) technologies are now significantly impacting the detection, identification and quantification of any known or novel virus present in a sample [6]. The availability of HTS technologies provides new opportunities for deep characterization of *Prunus* virome and to identify viruses potentially responsible for the diseases with unknown or poorly characterized etiology. A number of diseases of sweet cherry that were first described already in the 1940s are presumed to have a viral etiology based on the graft-transmissible nature of these diseases [7]. The rusty mottle group is a complex assemblage of diseases affecting sweet cherry that include cherry rusty mottle disease (CRMD), cherry necrotic rusty mottle disease (CNRMD), Frogmore canker, cherry bark blister and Lambert mottle [8]. Trees affected with CRMD exhibit chlorotic mottling of basal leaves that abscise prematurely while the remaining leaves become bright yellow or red as the season progresses. In contrast, CNRMD affected trees of some sweet cherry varieties show distinct angular necrotic leaf spots that turn to shot holes later in the season. In the U.S.A, it was observed that part of the buds and leaf spurs were killed, resulting in bare, rangy branches that were killed in more advanced stages of CNRMD and the cultivars ‘Lambert’, ‘Sam’, ‘Seneca’, and ‘Hudson’ had the most severe reaction [9]. Posnette and Cropley [10] reported CNRMD to be widespread and prevalent in English orchards with low productivity in the ‘Frogmore’, ‘Florence’, and ‘Noble’ cultivars. Trees infected with severe rusty mottle develop autumnal colours early so that 30% to 70% of leaf loss is observed by the time fruit ripening occurs [8]. The extent of mottling depends on the virus species and host cultivar. Some virus species cause dark red mottling while others may induce yellow or pale rusty pigmentation. Some commercial sweet cherry cultivars can be latent carriers of CRMD agent [11]. 

Different virus-like sequences have recently been reported to be associated with CRMD and CNRMD; they were identified as Cherry rusty mottle-associated virus (CRMaV) and Cherry necrotic rusty mottle virus (CNRMV), respectively [12], both belonging to *Betaflexiviridae* family. Within this family, different virus species are expected to have less than 72% nucleotide identity (or 80% amino acid identity of encoded proteins) in the coat protein (CP) or replicase/RNA dependent RNA Polymerase (*RdRp*) genes. Viruses from different genera usually have less than about 45% nucleotide identity in these genes [13]. Their discovery triggered the definition and proposal of a new genus, named *Robigovirus*, in the *Betaflexiviridae* family by the International Committee on Taxonomy of Viruses (ICTV) since 2016 [14]. The name suggested for the proposed genus is *Robigovirus* (derived from the Latin word “robigo,” meaning rust) and is chosen based on the symptoms associated with these viruses in sweet cherry cultivars. This genus also includes the cherry green ring mottle virus (CGRMV), another well-known *Prunus* virus [15]. The two species CNRMV and CGRMV are already accepted as species of the *Robigovirus* genus. 

The *Robigovirus* genus currently consists of five species represented by four viruses infecting *Prunus* spp. and one virus infecting palms. CRMaV and CNRMV are associated with rusty mottle symptoms, while cherry twisted leaf associated virus (CTLaV) is associated with a milder and delayed expression of rusty mottle symptoms [15]. Although the CGRMV infection can lead to leaf necrosis, leaf twisting and necrosis in fruits of sensitive cherries it is usually symptomless [16]. African oil palm ringspot virus (AOPRV) is a tropical virus infecting monocotyledonous palms causing ‘ringspot’ disease with a systemic yellowing, ringspots and elongated rings on leaflets and rachis of affected leaves, which eventually turned brown and died within a short period [17]. 

In Turkey, there has been some detection and characterization studies on commonly known cherry viruses like prune dwarf virus (PDV), prunus necrotic ringspot virus (PNRSV) and apple chlorotic leaf spot virus (ACLSV) [18,19] but no data has been reported so far for new emerging cherry viruses. In this study, we describe the discovery and the full genome sequencing of a new RNA virus tentatively named cherry virus Turkey (CVTR) belonging to the *Robigovirus* genus from cherries and the results of an epidemiological survey conducted in the most important cherry producing regions in Turkey. 

## 2. Results and Discussion

### 2.1. High Throughput Sequencing and Bioinformatics Analysis

The HTS yielded a total number of 8,344,550 reads (4,177,275 paired reads) ranging from 84 to 151 nucleotides. After quality trimming by BBDuck and Dedupe, 3,080,225 unique reads were obtained. The reads were assembled into 61,439 contigs summing 25,190,414 nucleotides. The maximal, minimal and median lengths were 13,954, 91 and 268 nucleotides, respectively. 

The TBLASTX analysis with a cutoff expected value (E-value) of 1 × 10^−5^ identified a contig of 8444 nucleotides with high homology (E-value of 0) to the cherry rusty mottle associated virus (CRMaV). The contig (hereafter called viral contig) had a match on 428 amino acids with an identity of 74% (315 identical sites). No other plant virus was identified in the generated contigs. Further confirmation was carried out by a BLASTX search on the non-redundant (nr) database (Table S1). Subsequently, 38 whole genome sequences of the five closest viruses (belonging to *Robigovirus, Betaflexiviridae*) were retrieved from The National Center for Biotechnology Information (NCBI) for further analysis. The sequences were aligned against the viral contig and the highest identity (57.1%) were found in CTLaV which was in agreement with BLASTX results (Table S2). 

**Figure 1:**
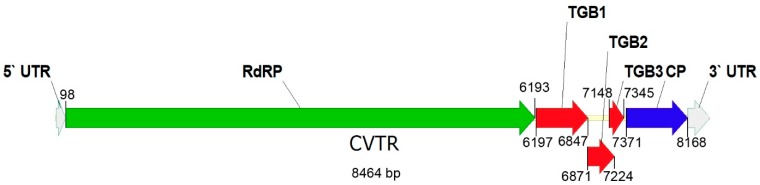
Genome organization of cherry virus Turkey (CVTR) as annotated from *Betaflexiviridae* reference sequences. The genome contains five ORFs. *RdRp*, replicase; *TGB*, triple gene block; *CP*, coat protein.

Conserved domains assessment of the viral contig revealed the presence of several plant viral domains, especially those of *Betaflexiviridae* (Table 1). Further annotation revealed that the viral contig has typical genome organization of *Betaflexiviridae* members (Figure 1). Distinctive properties of genera in the family *Betaflexiviridae* are given in Table S3 according to Adams et al. [13]. The comparisons of the viral contig with related viruses showed that it has a complete genome sequence for a virus belonging to *Betaflexiviridae*. It has five complete ORFs with size range close to the *Robigovirus* genus. Potexvirus characteristics are added for comparison since it has the same genome organization in *Alphaflexiviridae*. Properties of the viral contig are estimated by ProtParam software.

### 2.2. Whole-Genome Sequencing and Multiple Alignment Analyses

In order to validate the HTS results, overlapping primers along with 5′/3′ RACE (Table S4) were used to determine full-genome of the virus. This virus has a unipartite ssRNA genome which is 8464 nucleotides (nt) in length and codes for five ORFs. Additionally, two non-coding regions were found at the genome ends, 5′ UTR and 3′ UTR of 97 and 296 nt, respectively. The genome contains an open reading frame for *RdRp* comprising 6096 nt which codes for 2031 amino acids (232 kD) protein (Table S3). The *RdRp* gene of CVTR showed 51.9–58.7% and 43–53.1% identities at nt and amino acid (aa) levels, respectively with other robigoviruses (Table S5). As seen in Table 1, CVTR contains five domains in *RdRp*: (1) a methyltransferase domain (residues 43–352) which is found in a wide range of ssRNA viruses, including Hordei-, Tobra-, Tobamo-, Bromo-, Clostero- and Caliciviruses; (2) an RNA dependent RNA polymerase (*RdRp*-2 super family) domain (residues 1691–2012); (3) peptidase-C23 super family (residues 1045–1132); (4) viral helicase1 super family (residues 1226–1475); and (5) 2OG-Fe (II) oxygenase superfamily (residues 752–845). The genome sequences based on HTS and Sanger sequencing were deposited in GenBank under accession numbers MH177869 and MK600387, respectively.

Similar to other members of *Flexiviridae*, CVTR has three-partite movement proteins called triple gene block (*TGB*). The size of these three overlapping ORFs are as follows: *TGB1* corresponds to 675 nt which codes for a protein of 225 aa (25 kD). It shares 44.7–60% aa and 52.4–58.8% nt identity to the other five robigoviruses. *TGB2* has 351 nt (117 aa, 12.9 kD) and shares 39.8–44.4% sequence identities at the amino acid level, and 48.7–53% at the nt level to the above mentioned viruses. *TGB3* is 195 nt in length (65 aa, 7 kD) and shares 38–44.8% identities at the amino acid and 54.9–58.5% at nt levels, respectively. Finally, ORF 5 is 798 nt in length, encodes for a 268 aa coat protein (30 kD). A comparison of this ORF revealed that CVTR shares sequence identity to the other five viruses in a range of 45.3–50.6% at the amino acid and 52.4–57.6% at nt levels, respectively (Table S5). Among robigoviruses, CGRMV, CNRMV, CRMV and CTLaV encode five ORFs plus two additional ORFs, 2a and 5a, nested within ORF2 and ORF5, respectively [15]. Unlike the four cherry robigoviruses, CVTR lacks ORFs 2a and 5a therefore its genome organization is more similar to AOPRV due to no internal nested ORFs being detected in both viruses [20].

Four viruses belonging to *Betaflexiviridae* and infecting cherry have been already described and their genomes fully sequenced [15]. Two of them, the CGRMV and CNRMV have been classified in a new genus, together with two new species. When analyzing 24 complete genome sequences of the four virus species, an identical genome organization (replicase, *TGB* and coat protein) is observed. They fall into four distinct clades that correspond with distinct symptoms in cherry and woody indicator hosts [21]. This genome organization is shared with the *Foveavirus*, although the protein identity is lower than 40% with this genus. Moreover, phylogenetic analyses of the replicase genes of *Betaflexiviridae* viruses do not justify placing the cherry viruses in genus *Foveavirus*. Amino acid and nucleotide identities within and between the four closely-related cherry viruses, as a general rule, satisfy the molecular criteria used in the family that different species should have less than about 72% nt identity (or 80% aa identity) between their respective *CP* or replicase genes [13]. According to the species and genus demarcation criteria for *Betaflexiviridae*, cherry virus Turkey (CVTR) can be considered as a new species in genus *Robigovirus*.

### 2.3. Phylogenetic Analysis

**Figure 2:**
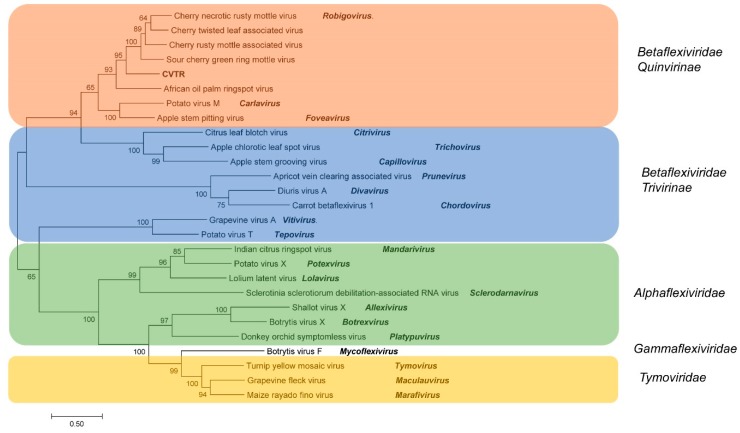
Molecular Phylogenetic analysis of full-genome Tymoviral reference sequences by Maximum Likelihood method based on the General Time Reversible model with a discrete Gamma distribution. The tree is drawn to scale, with branch lengths measured in the number of substitutions per site.

**Figure 3:**
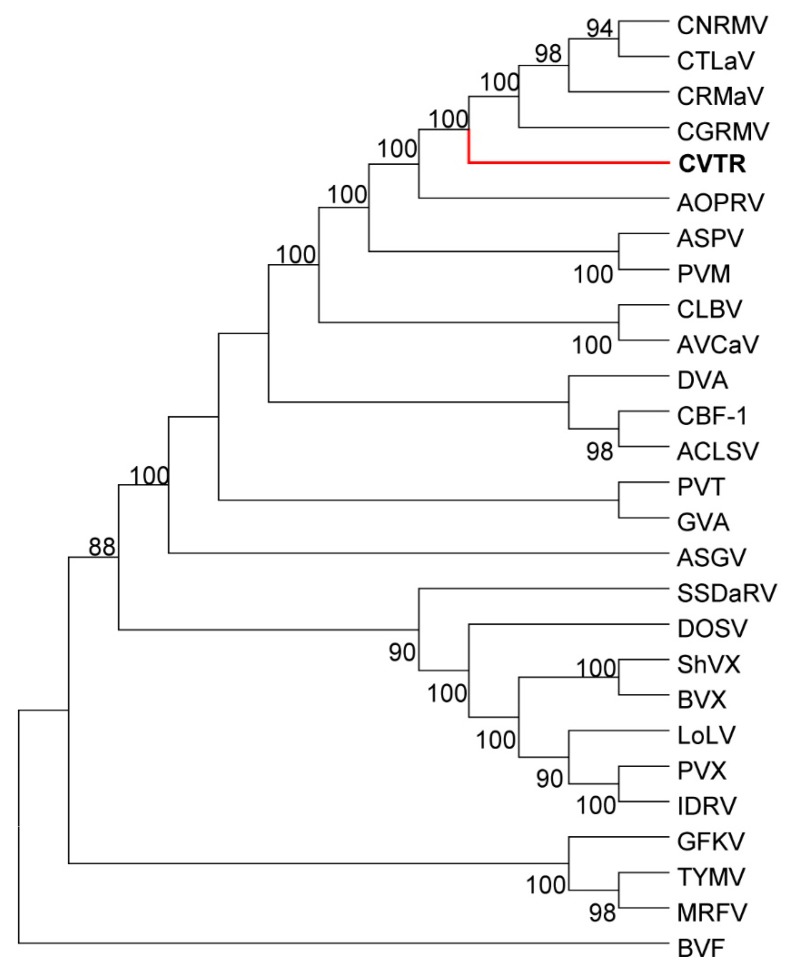
Phylogenetic tree reconstructed using the amino acid sequences of the *RdRp* ORF of the *Tymovirales* by ML method using LG+F substitution model with gamma distribution and invariant sites (LG+F+G+I model). Bootstrap values less than 60 are not shown. This tree robustly confirms (100% Bootstrap) that CVTR is distinct from its close related viruses and in line with the results obtained from the tree reconstructed from full genome sequences.

The phylogenetic tree reconstructed from full-genome reference sequences showed clustering of the CVTR into a group along with four robigoviruses (CTLaV, CNRMV, CRMaV and CGRMV), members of *Quinivirinae* subfamily, *Betaflexiviridae* family (Figure 2). Two closest species in *Quinvirinae*, a carlavirus and a foveavirus were placed in a distinct clade. In phylogenetic analyses of *RdRp* at aa level using the Maximum Likelihood method (ML) and LG+G model [22], CVTR was distinct from related viruses (Figure 3). Phylogenetic tree for *CP* aa sequences inferred by using the ML method based on the General Reverse Transcriptase + Freq. model [23] resulted in the same topology (Supplementary Materials, Figure S1). According to Villamor et al. [15] full genome sequences of CTLaV, CRMaV, CGRMV and CNRMV revealed segregation of sequences into four major clades with each clade represented by the following viruses: CTLaV (clade I), CNRMV (clade II), CRMaV (clade III), and CGRMV (clade IV). Comparative phylogenetic analysis consistently showed that CVTR is a new member of the *Robigovirus* genus, and particularly related to CTLaV, CRMaV, CGRMV and CNRMV, and to a lesser extent to AOPRV.

### 2.4. Transmission Electron Microscope (TEM) Analysis

**Figure 4:**
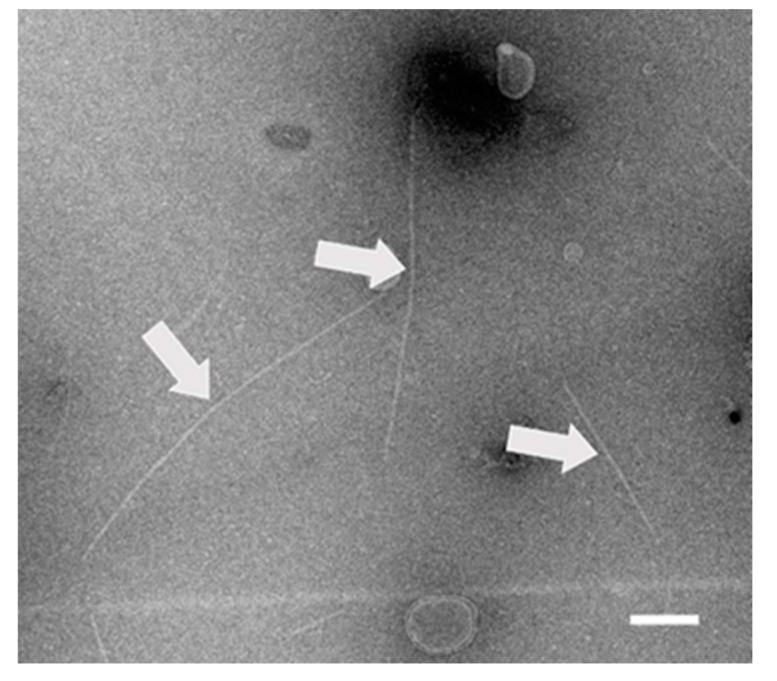
Filamentous particles (arrows) obtained after viral particle enrichment from cherry leaves. The scale bar represents 200 nm.

Viral particles were purified from leaves of the cherry tree, and analyzed by HTS. Electron micrographs showed filamentous particles (Figure 4), with a length ranging from 500 to 1400 nm (the most frequent length being in range of 1000–1200 nm) and a width of 11 nm. Virions in *Betaflexiviridae* family are flexuous filaments and usually 12–13 nm in diameter (range 10–15 nm) and from 600 to over 1000 nm in length, depending on the genus [24]. Due to the fact that *Robigovirus* genus was recently assigned, there are not many electron micrographs available to directly compare the particles of CVTR in this study. Transmission electron microscope observations of ultrathin sections of Kwanzan cherry trees affected by green ring mottle disease, caused by CGRMV, revealed the presence of flexuous, rod-shaped virions ranging from 1000 to 2000 nm in length and were 5–6 nm in diameter [25]. Beside this, direct observation of negatively stained leaf extracts prepared from AOPRV infected oil palms showed also the presence of filamentous virus-like particles c. 800 nm long and 15 nm in diameter [17]. All these findings together with molecular and phylogenetic data implies the association of filamentous particles and CVTR belong to the *Robigovirus* genus.

### 2.5. Field Surveys of the Novel Robigovirus Prevalence in Cherry Commercial Orchards

**Figure 5:**
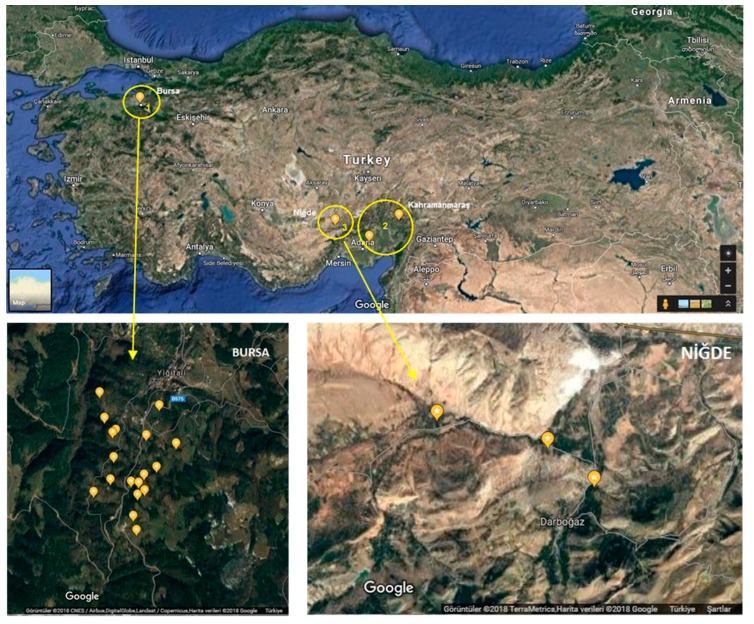
Map of the surveyed sweet cherry areas. The yellow circles in the upper map of Turkey show the positions of the surveyed provinces, which are expanded in the lower maps. Locations of positive samples for cherry virus Turkey (Bursa-Yiğitali and Niğde-Darboğaz) were placed on the map using a global positioning system and Google Earth.

In order to investigate the incidence of CVTR in four different provinces belonging to three geographical regions of Turkey with major relevance in cherry production (Bursa, Niğde, Kahramanmaraş and Adana), 156 samples were collected and tested by RT-PCR. The map of the surveyed areas and the locations of positive samples for CVTR are shown in Figure 5. 

**Figure 6:**
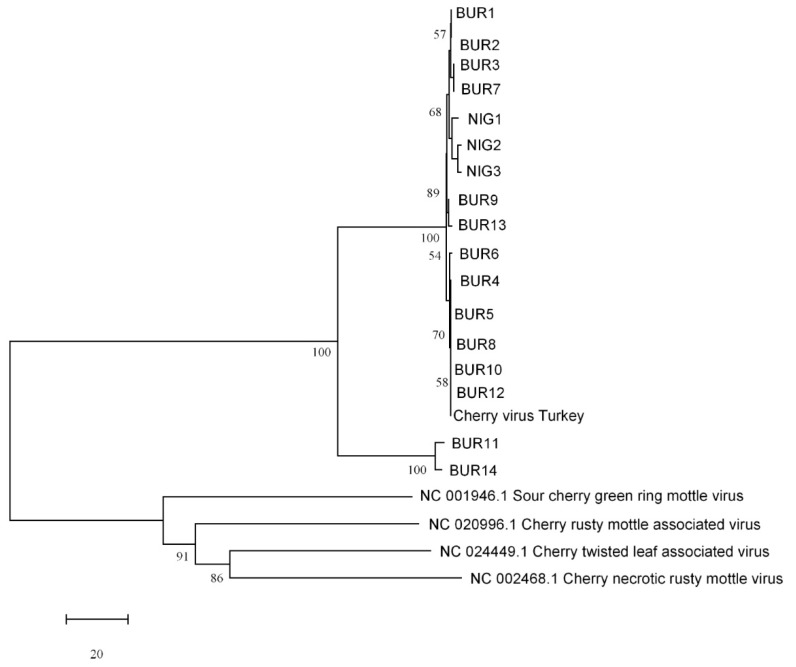
Phylogenetic tree reconstructed using the nucleotide sequences from the coat protein of cherry virus Turkey isolates (BUR1-14 and NIG1-3) and representative members of the genus Robigovirus. Tree was constructed by the neighbor-joining method and the statistical significance of branches was evaluated by bootstrap analysis (1000 replicates). Only bootstrap values above 50% are indicated. The scale bar represents 20% nucleotide divergence.

When two primer pairs 5436F/6031R (595 bp, *RdRp* region) and 7336F/8134R (828 bp, *CP* region), designed according to the sequence of CVTR genome (Table S4) were used in RT-PCR analysis, the *CP* region was successfully amplified from 17 samples while the *RdRp* region was successfully amplified from six of these samples. Among positive samples, 14 out of 43 were from the Bursa province where originally HTS analyzed sweet cherry sample was collected. Two sour cherry and one sweet cherry sample out of 81 tested were also found positive from the Niğde province, however no positive cherry sample was detected in Adana and Kahramanmaraş (*n* = 32). Seventeen amplicons from the *CP* region (MH986197-MH986213) and six amplicons from the replicase region (MH986214-MH986219) were sequenced and deposited in GeneBank (Table S6). The comparison of the partial CP nucleotide sequences showed that they shared 86.41% (BUR11) to 100 % (BUR5) identity with CVTR (Table S7). The phylogenetic tree, including the *CP* nucleotide sequences from *Robigovirus* members, confirmed a clear clustering of all CVTR isolates in the same clade (Figure 6). Among CVTR isolates, 15 of them were grouped together with CVTR full genome, while two divergent isolates (BUR11 and BUR14) were grouped into a different clade which was supported by 100% bootstrap value. Phylogenetic analyses of CVTR isolates based on the *RdRP* region resulted in similar topology with the *CP* tree and BUR11 and BUR14 isolates were distinct from the others (Figure S2). Multiple alignment of partial *RdRp* sequences showed that the isolates share 87% (BUR14) to 99% (BUR8) identity with the original CVTR isolate (Table S8). 

In summary, the prevalence of CVTR was 12% in tested samples collected from three different geographical regions of Turkey, but rose to 32% in the Bursa province only. CVTR was detected mainly in local cherry cultivar (cv. 0900 Ziraat) in Bursa (Marmara Region) and also in sour cherries (unknown cv.) (Niğde-Central Anatolia). We also tested the same 17 CVTR-positive samples for the presence of other prunus viruses (PDV, PNRSV, CVA, LChV1 and LChV2) some of which are already known to be widespread in Turkey [19]. Only five asymptomatic samples were found positive for PDV (results are not shown) while no positive sample was observed for the other viruses. It seems that CVTR infects cherry trees with or without eliciting obvious symptoms, but this data should be confirmed by bioassays in woody and possible herbaceous hosts in future studies. 

## 3. Materials and Methods 

### 3.1. Plant Material

The sample from one cherry plant showing slight chlorotic spots on the leaves grown in Bursa province, Turkey (isolate BUR12) was collected in May 2017 and submitted to high throughput sequencing of total RNA. In addition, 156 cherry samples were collected from four provinces, belonging to three geographical regions (Bursa-Marmara Region, Adana and Kahramanmaraş-Mediterranean Region and Niğde-Central Anatolia Region), where cherry production is economically important in Turkey. Among collected samples, only five sweet cheery plants in Bursa province were exhibiting some chlorotic spots and mottling.

### 3.2. RNA Extraction, Library Preparation and HTS

RNA extraction was carried out using RNeasy Plant Mini Kit (Qiagen) and the sample preparation buffer (RLT buffer) was supplemented with Plant RNA Isolation Aid (ThermoFisher Scientific). The RNA was treated with Turbo DNase (ThermoFisher Scientific) and concentrated by precipitation in sodium acetate and ethanol. After resuspending the extracted RNA, the quality and quantity were checked by Nanodrop (Thermofisher), Picogreen (Thermofisher) and 2100 Bioanalyzer System (Agilent). The ribosomal RNA was removed using the Ribo-Zero™ Plant Leaf Kit (Illumina) and the library was prepared using the TruSeq Stranded Total RNA Library Prep Kit (Illumina). The samples were sequenced on the Illumina Nextseq 500 platform with paired sequencing of 2 × 150 nt at the GIGA facilities of Liège University (Liege, Belgium).

### 3.3. Bioinformatic Analysis

The reads were trimmed for adapters and sorted by sample using Basespace (Illumina). The quality of sequences was further analyzed by FASTQC v0.11.7 [26]. The Geneious R10 software (https://www.geneious.com) was used for sequence analysis. BBDuck (version 36.92) plugin removed low quality reads using default settings. Furthermore, duplicated reads were removed by Dedupe plugin integrated into Geneious with a k-mer = 31. The trimmed reads were then de novo assembled into larger contigs by SPAdes [27] with a k-mer of 51. The generated contigs were further annotated using TBLASTX on the refseq database of viral nucleotides sequences downloaded from NCBI (ftp://ftp.ncbi.nlm.nih.gov/refseq/release/viral). A single hit was retrieved for each contig. Contigs with homologies with plant viruses were further analyzed by BLASTX on the non-redundant (nr) protein database from NCBI [28]. The contigs presenting high homology with plant viruses were mapped on the reference genome of closely related viral species using Geneious aligner (standard parameters, Geneious R10). Subsequently, the specified contigs were analyzed by ORF finder program and annotated. Conserved domain identification was carried out on the NCBI website using the Conserved Domain Search Service (CD-search) [29]. Complete annotation of the contig was carried out using Geneious live annotate and predict. Molecular mass of putative proteins was estimated using ProtParam software (https://web.expasy.org/protparam/).

### 3.4. Determination of the Full-Length Genome Sequences

After the assembly of HTS data, several overlapping primer pairs were designed to validate deep sequencing results by Sanger sequencing. The sequences of the primers used to complete the CVTR genome sequence, are presented in Table S4. The two genome ends were determined by a Rapid Amplification of Complementary DNA Ends (RACE) kit (SMARTer^®^ RACE 5′/3′, Clontech) according to manufacturer’s instructions [30]. Briefly, first strand cDNA synthesis carried out by SMARTScribe RT using a modified oligo (dT) primer and SMARTer II A Oligonucleotide. Following reverse transcription, 5′ and 3′ RACE PCR reactions were accomplished using four gene specific primers (GSP for direct PCR and NGSP for nested-PCR) and two universal long and short primers. All amplified fragments were directly sequenced by Iontek, Istanbul, Turkey. 

### 3.5. Survey for Presence of the Virus by RT-PCR

For the survey of virus presence in cherry orchards, extraction of total nucleic acids (TNAs) was performed by using RNeasy Plant Mini Kit (Qiagen, Hilden, Germany). The virus was detected by RT-PCR. First 5 µL of RNA were reverse transcribed using random hexamer primer (Thermo Fisher Scientific) and cDNA was amplified using detection primer pairs which amplifies coat protein and *RdRp* genes (listed in bold in Table 1) in a 25 µLreaction volume containing 2 µL of the RT mixture, 1.5 mM MgCl_2_, 0.2 mM each dNTP, 0.4 µM each primer, Fermentas reaction buffer and 1 U Taq-polymerase. After an initial PCR activation step at 95 °C for 5 min, 40 cycles of 95 °C for 1 min, 56–68 °C for 1 min (the annealing temperature was changed according to the primer used) 72 °C for 1 min were performed, followed by a final extension at 72 °C for 10 min. The amplified fragments were visualized by agarose gel electrophoresis and their specificity confirmed by Sanger sequencing (Iontek, Istanbul).

### 3.6. Phylogenetic Analysis

The phylogenetic tree was reconstructed from nucleotide reference sequences of Tymovirales listed in ViralZone (https://viralzone.expasy.org/294). Multiple alignments of nucleotide sequences or of deduced amino acid sequences were performed using the ClustalW program implemented in MEGA v7 [31]. Phylogenetic trees for full-genome, for *RdRp* and *CP* genes, were constructed from aligned sequences using the maximum likelihood by best fitting methods and bootstrapping evaluation of branching validity. Nucleotide and amino acid identities were determined by pairwise sequence alignment with Muscle program embedded in Geneious [32] and EMBOSS Needle (https://www.ebi.ac.uk/Tools/psa/emboss_needle/).

### 3.7. Virus Purification and Transmission Electron Microscopy (TEM) Analysis

Virus particles were purified from mature leaves as described previously [33]. The enriched fractions were negatively stained in 2% uranyl acetate and examined for the presence of viral particles using a Tecnai Spirit BioTWIN transmission electron microscope (FEI, Hillsboro, OR, USA).

## 4. Conclusions

We describe a new virus, designated cherry virus Turkey (CVTR), identified from a slightly symptomatic cherry tree by HTS analysis. Considering the accepted species demarcation molecular criteria for the family *Betaflexiviridae*, which must have 72% nt identity (or 80% aa identity) in the replicase and *CP* genes [13], CVTR should be considered as a novel species in the genus Robigovirus. According to our surveys, this virus is widespread in main commercial cherry orchards located in two different geographical regions of Turkey, even though there is no clear association between the presence of the virus and the symptoms. Furthermore, the genetic diversity among CVTR isolates was very high and we found two divergent isolates which could be of importance for detection and certification programs. As proposed in a recently published framework [34], investigating the epidemiology and pathogenicity of CVTR will also be a priority to characterize this new virus species and evaluate the risk it can pose to cherry production in Turkey.

## Figures and Tables

**Table 1:** List of conserved domains of the viral contig predicted by NCBI Conserved Domain Search.

Name	Accession	Description	Interval	E-value
**Vmethyltransf**	pfam01660	Viral methyltransferase	208–1137	1.24 × 10^−77^
**2OG-FeII_Oxy super family**	cl21496	This family contains AlkB.	2335–2616	8.63 × 10^−7^
**Peptidase C23 super family**	cl05111	Carlavirus endopeptidase	3214–3477	1.21 × 10^−13^
**Viral helicase1 super family**	cl26263	Viral (Superfamily 1); helicase activity	3757–4506	1.44 × 10^−8^
	pfam01443	Viral (Superfamily 1); helicase and NTPase activities	6253–6840	4.10 × 10^−39^
***RdRP*** ***2* super family**	cl03049	RNA dependent RNA polymerase	5152–6117	3.13 × 10^−40^
**Plant vir prot super family**	cl03157	Plant viral movement protein; potexviruses, hordeiviruses and carlaviruses	6864–7184	2.42 × 10^−18^
**7kD coat**	pfam02495	7kD viral coat protein; carlaviruses and Potexviruses	7135–7317	1.18 × 10^−9^
**Flexi *CP* super family**	cl02836	Viral coat protein; potexviruses and carlaviruses.	7592–7999	4.42 × 10^−46^

## Supplementary Materials

The following information is available online at https://www.mdpi.com/2076-0817/8/2/57/s1, Figure S1: Molecular Phylogenetic analysis of *CP* aa sequences by Maximum Likelihood method based on the General Reverse Transcriptase + Freq. model; Figure S2. Phylogenetic tree reconstructed using the nucleotide sequences from the *RdRp* region of Cherry virus Turkey isolates; Table S1. BLASTX search of the viral contig on the nr database; Table S2: Multiple Genome Alignment of the viral contig vs. five most related *Robigovirus* species; Table S3. Distinctive properties of genera in the family *Betaflexiviridae*; Table S4. Primers used in this study to complete the genome sequence of the CVTR isolate. The primer pairs in bold have been used for the survey of commercial orchards; Table S5. Characteristics of the species belong to *Betaflexiviridae* used for comparison of cherry virus Turkey (CVTR). Values are percentage identity of nucleotides and amino acids pairwise alignment between given viruses and CVTR by EMBOSS Needle; Table S6. List of cherry sources found infected by Cherry virus Turkey in the present study; Table S4. Identity matrix of *RdRp* region for different isolates of CVTR; Table S7. Identity matrix of *CP* region for different isolates of CVTR; Table S8. Identity matrix of *RdRp* region for different isolates of CVTR.
