# Supplementary material for: Identification and Characterization of a Novel Robigovirus Species from Sweet Cherry in Turkey
Source: Pathogens. 2019 Apr 27;8(2):57. doi: 10.3390/pathogens8020057 (PMC6631170; doi:10.3390/pathogens8020057)
Supplement: Supplementary file 1 [file pathogens-08-00057-s001.pdf]

Supplementary Materials

Table S1. BLASTX search of the viral contig on nr database.

Virus species	Acronym	Identical sites	Identity %
cherry twisted leaf associated virus	CTLaV	1120/2081	54
cherry rusty mottle associated virus	CRMaV	1108/2068	54
cherry necrotic rusty mottle virus	CNRMV	1096/2081	53
cherry green ring mottle virus	CGRMV	1095/2065	53
african oil palm ringspot virus	AOPRV	591/1141	52
rubus canadensis virus 1	RuCV-1	550/1325	42
asian prunus virus 1	APV-1	554/1314	42
asian prunus virus 2	APV-2	546/1314	42
asian prunus virus 3	APV-3	546/1313	42
cherry virus B	CVB	551/1317	42
cherry mottle leaf virus	CMLV	308/815	38
cherry virus A	CVA	310/838	37

Table S2. Multiple Genome Alignment of the viral contig vs. five most related *Robigovirus* species.

Virus species	Acronyme	Accession Number	Length (bp)	Identical Sites (bp)	Identical Sites%
**Cherry twisted leaf associated virus**	CTLaV	NC 024449	8,431	5003	57.1
		KF030846	8,426	4784	54.8
		KF030848	8,428	4,757	54.6
		KF030880	8,428	4844	55.5
		KF030859	8,434	4839	55.5
		KF030865	8,433	4,842	55.6
		KF030878	8,434	4,782	54.9
		KP258177	8,428	4,852	55.6
		KF030873	8,425	4,788	55
**Cherry necrotic rusty mottle virus**	CNRMV	NC_002468	8,432	4,879	55.3
		KY310583	8,426	4,816	54.9
		EU188439	8,429	4,841	55.2
		KR820549	8,428	4,823	55
		EU188438	8,430	4,849	55.3
		KY178274	8392	4816	55
		KF030832	8428	4,801	54.8
		LC064751	8124	4,697	55.9

**Cherry rusty mottle associated virus**	CRMaV	NC 020996	8,397	4,976	56.9
		KX389311	8,377	4,832	55.7
		KP258176	8,402	4,728	54.2
		KF356396	8,394	4,835	55.7
		KC218927	8,398	4,762	54.9
		KF030869	8404	4,828	55.6
		KF030849	8404	4,844	55.8
		KF030850	8404	4,828	55.6
		KF030870	8405	4,832	55.6
**Sour cherry green ring mottle virus**	SCGRMV	NC 001946	8,372	4,914	56.1
	CGRMV	KR820548	8,371	4,793	55.3
		KC218931	8,375	4818	55.6
		JX501671	8,377	4808	55.4
		JX501670	8,373	4,776	55.1
		AJ291761	8,376	4800	55.4
		KY774448	8304	4753	54.7
		KY178276	8431	4780	55.1
		KY178275	8353	4,775	55.1
		KY178277	8348	4799	55.4
		LC064752	8151	4,686	55.9
**African oil palm ringspot virus**	AOPRV	NC_012519	7,781	4,488	51.6

Table S3. Distinctive properties of genera in the family Betaflexiviridae, molecular weight of each protein presented in K (Adopted from Adams et al., 2012).

**Genus**	**Species**	**Virion length (nm)**	**ORFs**	**Rep**	**TGB1**	**TGB2**	**TGB3**	**CP**
**Robigovirus**	CNRMV	800	5	231	25	12	7	30
	CGRMV	N.D.	5	229	25	12	7	30
	CRMaV		5	229	25	12	7	30
	CTLaV		5	230	25	12	7	30
	AOPRV		5	209	25	12	7	29
	CTVR contig		5	232	25	12	7	30
**Foveavirus**	ASPV	800	5	230-250	25	12	7	28-44
**Carlavirus**	PVM	610-700	6	215-225	25	12	7	32-36
**Potexvirus**	PVX	470–580	5	165	25	12	8	18–27

Table S4. Primers used in this study to complete the genome sequence of the CVTR isolate. The primer pairs in bold have been used for the survey of commercial orchards.

Primer Name	Sequence (5′ – 3′)	Amplicon size	Location
66F	CCTACAACGTTTACCCATGGC	694 bp	66-759
715R	CCAGGACCTCAGGTGGAAAGAC		
609F	GTATGATTGAACTGCACTATTGG	727 bp	609-1335
1335R	CTTAACAGACTCAATGAATTTCC		
1165F	GAGGACTTTGTTGAGTTCCTGC	615 bp	1165-1783
1783R	TCCTTAGTCTGGAGCTAACCTC		
1644F:	AAGAATCATGCTAGTGAGGGG	680 bp	1644-2323
2323R:	CCTTGCTCATAACATCAGGCCA		
2193F	AGGGCTTGATTTCTCAAAAGG	718 bp	2193-2910
2910R	AATGCTAGACACAGCATGCCA		
2795F	ATGAAGTGATGAATGGGTGTGC	705 bp	2795-3499
3499R	CTGAAGTCCTAGAATTGGGGC		
3382F	CAGGCAAGCATTTTTGATGG	693 bp	3382-4074
4074R	ACTTTGTGCAGGGTCCCCCAT		
3382F	CAGGCAAGCATTTTTGATGG	1309 bp	3382-4691
4691R	CTCTCTTCCCTATCCATTTCATC		
3985F	CAGCTCTTCCCACCTGGGTA	707 bp	3985-4691
4691R	CTCTCTTCCCTATCCATTTCATC		
4588F	GGGAGGGCTAGCGTTAATGA	875 bp	4588-5460
5460R	CTCATCAAAATTCTTTCCAGAGTG		
**5436F**	CCACTCTGGAAAGAATTTTGA	595 bp	5436-6031
**6031R**	GCCTCTCACCTAAATTATAGGC		
5880F	CTGCGGCTGGTGCATTGGGAG	337 bp	5880-6216
6216R	GCCTGCCCTGATCAGCTCCTC		
6005F	CCTATGCCTATAATTTAGGTGA	1334 bp	6005-7339
7339R	TCATAAGGCCTCAACCTAAGC		
**7336F**	CCTTACGCGCATGGCTTAGGTT	828 bp	7306-8134
**8134R**	CTGATTGATGGCCTTGATGGG		
8038F	CCGAGATGAGCTCAAGAGAGG	313 bp	8038-8350
8350R	AGCTAATGAGTAAGCAAGCCATA		
GSP1	CCAACCAGGACCTCAGGTGGAAAGA	5'RACE	
NGSP1	TCTCGGGAAGTGACGCAACGATTGAC		
GSP2	GGCTCCAGCGAGTACACCAAATTCT	3'RACE	
NGSP2	GCGTAACAACCCTGAGGCAGTTCTG		

Table S5. Characteristics of the species belong to *Betaflexiviridae* used for comparison of cherry virus Turkey (CVTR). Values are percentage identity of nucleotides and amino acids pairwise alignment between given viruses and CVTR by EMBOSS Needle.

Virus	Access. no.	RdRp			TGB1			TGB2			TGB3			CP		
		nt Identity %	aa Identity %	aa similarity %	nt Identity %	aa Identity %	aa similarity %	nt Identity %	aa Identity %	aa similarity %	nt Identity %	aa Identity %	aa similarity %	nt Identity %	aa Identity %	aa similarity %
**CTLaV**	NC 024449	58	53	68	58	58	73	53	44	58	52	44	64	52	50	64
**CNRMV**	NC 002468	57	52	68	57	59	73	49	39	53	51	42	56	53	49	63
**CRMaV**	NC 020996	58	53	69	54	59	72	52	43	53	53	41	59	57	50	64
**CGRMV**	NC 001946	55	53	69	55	60	72	50	40	49	58	41	58	53	49	66

**AOPRV**	AY072921	51	43	58	52	44	61	48	39	52	54	38	52	54	45	58
**ASPV**	NC003462	50	35	50	44	39	55	50	30	43	46	34	51	36	17	28
**PVM**	NC001361	49	33	47	41	31	46	47	36	43	52	28	39	42	23	36
**PVX**	NC011620	40	19	32	47	31	47	48	32	41	39	23	40	44	31	46

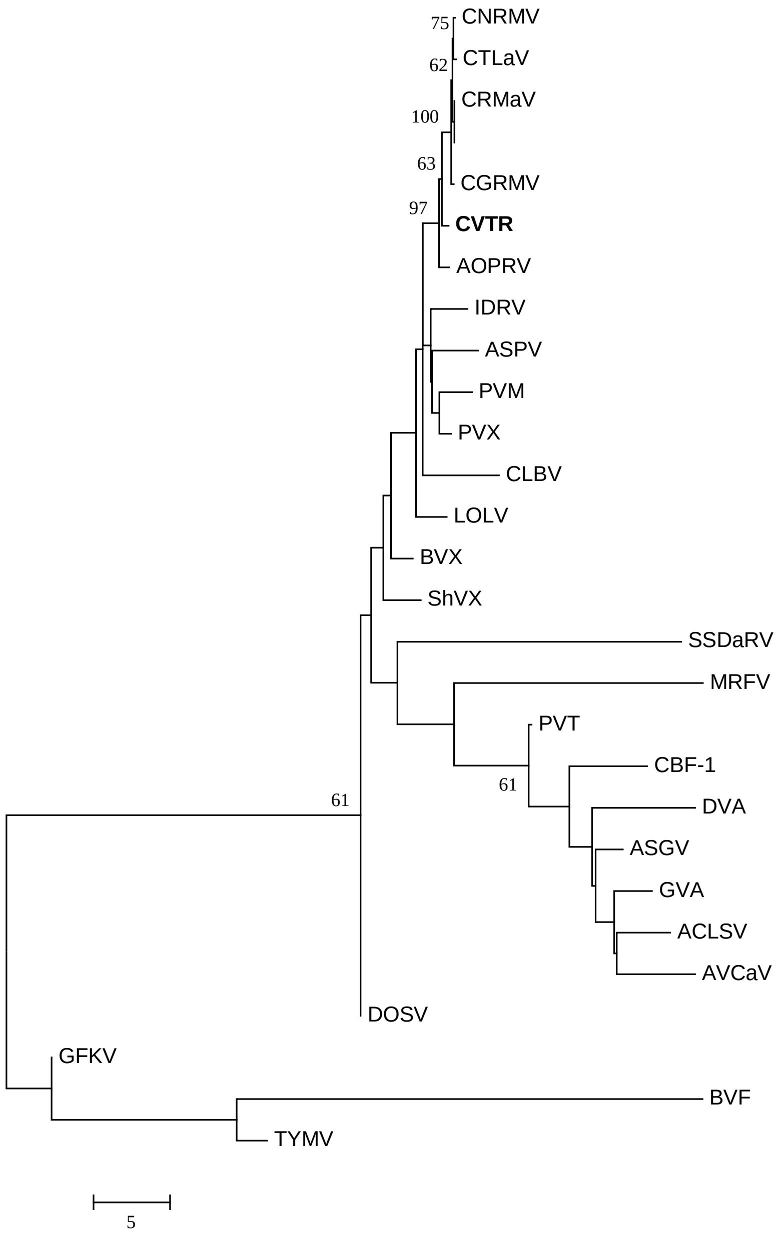

Figure S1. Molecular Phylogenetic analysis of CP aa sequences by Maximum Likelihood method based on the General Reverse Transcriptase + Freq. model. Bootstrap values less than 60 are not shown.

Table S6. List of cherry sources found infected by Cherry virus Turkey in the present study.

Source	Host	Location	Accession numbers for CP gene	Accession numbers for partial replicase gene
BUR1	Sweet cherry	Bursa	MH986197	-
BUR2	Sweet cherry	Bursa	MH986198	-
BUR3	Sweet cherry	Bursa	MH986199	-
BUR4	Sweet cherry	Bursa	MH986200	-
BUR5	Sweet cherry	Bursa	MH986201	-
BUR6	Sweet cherry	Bursa	MH986202	-
BUR7	Sweet cherry	Bursa	MH986203	MH986214
BUR8	Sweet cherry	Bursa	MH986204	MH986215
BUR9	Sweet cherry	Bursa	MH986205	MH986216
BUR10	Sweet cherry	Bursa	MH986206	-
BUR11	Sweet cherry	Bursa	MH986207	-
BUR12	Sweet cherry	Bursa	MH986211	MH986217
BUR13	Sweet cherry	Bursa	MH986212	MH986218
BUR14	Sweet cherry	Bursa	MH986213	MH986219
NIG1	Sweet cherry	Niğde	MH986208	-
NIG2	Sour cherry	Niğde	MH986209	-
NIG3	Sour cherry	Niğde	MH986210	-
CVTR	Sweet cherry	Bursa	MH177869 (Complete genome)	

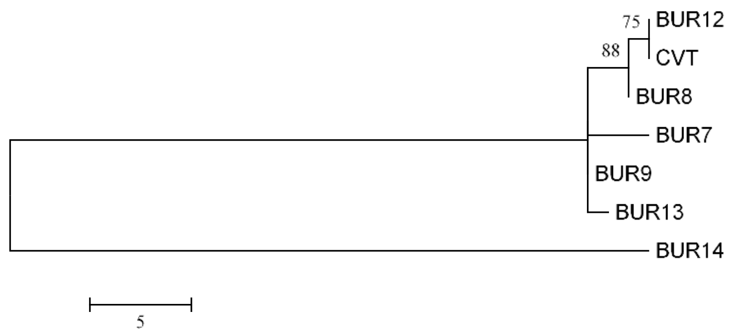

Figure S2. Phylogenetic tree reconstructed using the nucleotide sequences from the RdRp region of Cherry virus Turkey isolates (BUR7, 8, 9, 12, 13, 14). Tree was constructed by the neighbor-joining method and the statistical significance of branches was evaluated by bootstrap analysis (1000 replicates). Only bootstrap values above 50% are indicated. The scale bar represents 5% nucleotide divergence.

Table S7. Identity matrix of CP region for different isolates of CVTR.

	CVTR	BUR1	BUR2	BUR3	BUR4	BUR5	BUR6	BUR7	BUR8	BUR9	BUR10	BUR11	BUR13	BUR14	NIG1	NIG2	NIG3
CVTR		99.6	99.6	99.3	99.9	100.0	99.6	99.4	99.4	97.1	99.4	86.4	98.9	86.6	98.1	98.7	99.0
BUR1	99.6		100.0	99.7	99.4	99.6	99.1	99.8	98.9	97.3	98.9	86.6	99.0	86.7	98.3	98.9	99.1
BUR2	99.6	100.0		99.7	99.4	99.6	99.1	99.8	98.9	97.3	98.9	86.6	99.0	86.7	98.3	98.9	99.1
BUR3	99.3	99.7	99.7		99.1	99.3	98.9	99.9	98.6	97.1	98.6	86.3	98.7	86.4	98.3	99.1	99.4
BUR4	99.9	99.4	99.4	99.1		99.9	99.4	99.2	99.4	97.0	99.4	86.3	98.7	86.4	98.0	98.6	98.9
BUR5	100	99.6	99.6	99.3	99.9		99.6	99.4	99.4	97.1	99.4	86.4	98.9	86.6	98.1	98.7	99.0
BUR6	99.6	99.1	99.1	98.9	99.4	99.6		98.9	99.2	96.9	99.1	86.8	98.4	87.0	97.7	98.3	98.6
BUR7	99.4	99.8	99.8	99.9	99.2	99.4	98.9		98.7	97.1	98.7	86.3	98.8	86.5	98.2	99.1	99.4
BUR8	99.4	98.9	98.9	98.6	99.4	99.4	99.2	98.7		96.6	99.1	86.1	98.3	86.3	97.7	98.4	98.4
BUR9	97.1	97.3	97.3	97.1	97.0	97.1	96.9	97.1	96.6		96.6	86.9	97.4	87.1	96.6	96.4	96.6
BUR10	99.4	98.9	98.9	98.6	99.4	99.4	99.1	98.7	99.1	96.6		86.2	98.3	86.3	97.7	98.4	98.4
BUR11	86.4	86.6	86.6	86.3	86.3	86.4	86.8	86.3	86.1	86.9	86.2		87.4	99.3	86.7	85.7	85.8
BUR13	98.9	99.0	99.0	98.7	98.7	98.9	98.4	98.8	98.3	97.4	98.3	87.4		87.6	97.6	98.0	98.1
BUR14	86.6	86.7	86.7	86.4	86.4	86.6	87.0	86.5	86.3	87.1	86.3	99.3	87.6		86.8	85.8	86.0
NIG1	98.1	98.3	98.3	98.3	98.0	98.1	97.7	98.2	97.7	96.6	97.7	86.7	97.6	86.8		98.1	98.3
NIG2	98.7	98.9	98.9	99.1	98.6	98.7	98.3	99.1	98.4	96.4	98.4	85.7	98.0	85.8	98.1		99.4
NIG3	99.0	99.1	99.1	99.4	98.9	99.0	98.6	99.4	98.4	96.6	98.4	85.8	98.1	86.0	98.3	99.4	

Table S8. Identity matrix of RdRp region for different isolates of CVTR.

	BUR7	BUR8	BUR9	BUR13	BUR14	CVT
**BUR7**		98.68	97.37	98.77	86.82	98.77
**BUR8**	98.68		97.24	98.68	87.17	99.39
**BUR9**	97.37	97.24		97.72	86.73	97.37
**BUR13**	98.77	98.68	97.72		87.17	98.95
**BUR14**	86.82	87.17	86.73	87.17		86.99
**CVT**	98.77	99.39	97.37	98.95	86.99	
